# Systematic Review and Meta-analysis: Use of Statins Is Associated with a Reduced Incidence of Oesophageal Adenocarcinoma

**DOI:** 10.1007/s12029-017-9983-0

**Published:** 2017-07-10

**Authors:** Tom Thomas, Yoon Loke, Ian L. P. Beales

**Affiliations:** 1 Norwich Medical School, University of East Anglia, Norwich, NR4 TJ UK; 2 Department of Gastroenterology, Norfolk and Norwich University Hospital, Norwich, NR4 7UY UK

**Keywords:** Barrett’s oeosphagus, Chemoprevention, Cancer risk, Oesophageal carcinoma, Statins

## Abstract

**Purpose:**

Laboratory studies have suggested that statins may have useful anti-cancer effects against Barrett’s epithelial cancer lines. A variety of effects have been reported in clinical studies.

**Methods:**

We performed a systematic review and meta-analysis of the association between statin use and the development of oesophageal cancer. Multiple databases were searched for studies reporting the association of statin use and oesophageal cancer. Meta-analysis on the relationship between statin use and cancer incidence was performed.

**Results:**

Twenty publications met eligibility criteria, yielding 22 datasets for meta-analysis. All were observational studies. Population-level studies included 372,206 cancer cases and 6,086,906 controls. Studies examining adenocarcinoma development in Barrett’s oesophagus included 1057 cancers and 17,741 controls. In patients with Barrett’s oesophagus, statin use was associated with a reduced incidence of adenocarcinoma (pooled adjusted odds ratio (OR) 0.59 (95% confidence intervals 0.50–0.68)), with no heterogeneity between 11 studies. Population-based studies demonstrated more heterogeneity but showed that statin use was associated with a lower incidence of both oesophageal adenocarcinoma (OR 0.57 (0.43–0.76)) and all oesophageal cancers (OR 0.82 (0.7–0.88)). Information on statin type, dose, and duration was reported too infrequently for statistical analysis but individual studies showed a tendency to a dose- and duration-dependant decrease in cancer incidence.

**Conclusions:**

Statin use is associated with a significantly lower incidence of oesophageal adenocarcinoma. This is seen in both Barrett’s cohorts and general populations. Further studies should focus on drug, dose, and duration and the interaction with other risk and preventative factors.

**Electronic supplementary material:**

The online version of this article (doi:10.1007/s12029-017-9983-0) contains supplementary material, which is available to authorized users.

## Introduction

Oesophageal carcinoma (OC) affects 450,000 people worldwide and is the sixth leading cause of cancer-related mortality in the world [1]. The two most common histological subtypes, squamous cell carcinoma (OSC) and adenocarcinoma (OAC), together make up approximately 98% of this number. Global epidemiological studies show that whilst global incidence of OSC is greater than OAC, the incidence of OAC is now greater than OSC in the developed countries of the West such as the UK, the Netherlands, and the USA [2, 3]. Currently, prognosis for oesophageal cancer remains one of the poorest amongst all cancers, with 5-year survival rates around 15% [3, 4]. This is largely due to the proportion of patients presenting at advanced disease stages. In OAC in particular, this has led to management strategies revolving around monitoring risk factors, most important being Barrett’s oesophagus (BO). To this effect, endoscopic surveillance as well as ablative procedure, principally radiofrequency ablation (RFA), has been developed [5]. Although surveillance and intervention strategies do seem to reduce the incidence of OAC, RFA is not without costs and complications [5].

However, due to the generally asymptomatic nature of BO, most cases of OAC arise outside surveillance programs. This is an area where chemopreventative strategies could make a difference to the general population. In the past 10 years, experimental evidence has shown that statins [hydroxymethlyglutaryl-CoA reductase inhibitors (HMG-CoA inhibitors)] could potentially play a role in reducing progression of BO to OAC [6, 7]. The mechanisms proposed include upregulation of pro-apoptotic proteins such Bad and Bax, as well as reduced cell proliferation and enhanced apoptosis through reduced activation of signalling G-proteins as a consequence of reduced levels of melavonate due to inhibition of the HMG-CoA reductase [6, 7]. Through these mechanisms and possibly other mechanisms, statins attenuate the malignant behaviour of OAC cell lines [6–10]. Evidence from a variety of clinical studies supports this hypothesis. Four years ago, two separate meta-analyses using slightly different methods came to almost identical conclusions: statin use was associated with a significant 43% reduction in the incidence of OAC in Barrett’s cohorts and a smaller [19%] reduction in the incidence of all oesophageal cancers in population studies [11, 12]. However, these studies highlighted areas of lack of information including the precision of the inverse association with statin use, effects of confounding risk factors, and effects in different populations. Since the publication of these meta-analyses, multiple additional studies have been published and we have performed an updated systematic review and meta-analysis examining the association between statin use and OAC with the aim of further refining the association and in particular defining knowledge gaps that could be beneficially be the target of future studies [12]. The inclusion of more recent data has enabled us to specifically examine the association between statin use and oesophageal cancer in three categories: statin use in relation to malignant progression to OAC in Barrett’s oesophagus cohorts, the association between statin usage and OAC in a population cohorts, and the association between statin usage and all oesophageal cancers on a population scale.

## Methods

This systematic review was performed according to the Preferred Reporting Items for Systematic reviews and Meta-Analyses Guidelines (PRISMA guidelines) and as previously described [12, 13].

### Selection Criteria

Eligible study design characteristics included randomised controlled trials, observational studies (cohort and case-control design) that met the following inclusion criteria: statin exposure status with reliable evidence through record linkage or otherwise, reported disease incidence (oesophageal adenoncarcinoma or high-grade dysplasia, or oesophageal cancer), and provision of hazard ratio (HR), odds ratio (OR), or relative risk (RR), and in the case of their absence, provision of data for their calculation.

### Data Sources and Search Strategy

A systematic literature search of Medline, Embase, Web of Science, Cochrane database, and Google Scholar from inception until 4th February 2017 was performed in order to identify all relevant studies that investigated oesophageal cancer incidence in statin users. A combination of keywords and medical subject heading terms were used including “oesophageal neoplasm”, “Barrett’s oesophagus”, “oesophageal adenocarcinoma”, “statin”, and “hydroxymethylglutaryl-CoA reductase inhibitor”. Two investigators (Thomas T, Beales ILP) then independently reviewed and excluded articles that were not relevant to the research topic. A manual search of reference lists as well as relevant review articles was undertaken to screen for potential articles.

### Data Extraction and Quality Assessment

Data was extracted from the selected studies into a standardised table by the two chief investigators. This table is provided in Table 1. Data was organised into the three subgroups being examined. Information regarding duration and dose of statin use was also extracted alongside number of statin/non-statin users to further assess the impact of statins. Unadjusted and adjusted risk ratios were also extracted where available. We checked the validity of the included studies based on possibility of confounding and potential for misclassification of tumour pathology and/or drug exposure. Risk of bias assessment was focused on the selection of participants, comparability of cases and controls (with any adjustments for confounding), and methods used in ascertaining drug exposure and outcomes. The quality of all studies was assessed using the Newcastle-Ottawa Quality Assessment Scales by two investigators independently using the star-rated system as previously described [12, 34]. Any discrepancies in scoring were then resolved through combined reassessment and consensus by all authors. The final quality assessment scores are listed in Table 1.

**Table 1:** Characteristics of included studies

First author [ref]		Location, setting	Study design	Total number of patients	Primary outcome of interest	Patient on statins		Patient not on statins		Variables adjusted for	Newcastle-Ottawa score
						No. of EAC/EC cases	No. of non-EAC/EC cases	No. of EAC/EC cases	No. of non-EAC/EC cases		
Nguyen 2010 [14]		US; multicentre, hospital-based	Retrospective cohort	344 patients OAC 33	Incidence of OAC in Barrett’s cohort	6	63	27	188	1, 2, 7, 9	8^a^
Nguyen 2009 [15]		US; multicentre, hospital-based	Nested case control	812 patients OAC 116, BO 696	Incidence of OAC in Barrett’s cohort	41	336	75	360	1, 2, 3	9^a^
Kastelein [16]		The Netherlands; multicentre, hospital-based	Prospective cohort	570 patients HGD/OAC 38	Incidence of OACC in Barrett’s cohort	9	200	29	332	1, 2, 7, 8, 9	9^a^
Kantor [17]		US; hospital-based	Prospective cohort	411 patients OAC 56	Incidence of OAC in Barrett’s cohort	6	50	50	305	1, 2, 4, 9	9^a^
Beales 2012a [18]		UK; hospital-based	Case control	255 patients OAC 85, BO 170	Incidence of OAC in Barrett’s cohort	17	60	68	110	1, 2, 4, 5, 6, 8	8^a^
Krishnamoorthi [19]		US; population-based	Cohort	9660 patients OAC 103	Incidence of OAC in Barrett’s cohort	NR	NR	NR	NR	1, 2, 4, 6, 9	8^a^
Iyer [20]		UK; population-based	Cohort	NR	Incidence of OAC in Barrett’s cohort	NR	NR	NR	NR	NR	6^a^
Agrawal [21]		US; hospital-based	Case control	583 patients	Incidence of OAC in Barrett’s cohort	55	307	60	161	1, 4, 5, 6, 9	7^a^
Masclee [22]		UK and the Netherlands; multicentre, population-based	Nested case control	777 patients OAC 45, BO 732	Incidence of OAC in Barrett’s cohort	12	253	33	479	None listed	7^a^
Cooper [23]		UK; population-based	Nested case control	3749 patients OAC 55	Incidence of OAC in Barrett’s cohort	18	1124	37	2570	1, 2, 4	8^a^
Nguyen 2015 [24]		US; population-based	Nested case control	1167 patients OAC 311, BO 856	Incidence of OAC in Barrett’s cohort	125	462	186	394	1, 4, 6, 9	9^a^
Kaye [25]		UK; population-based	Case control	530 patients OC 100, control 430	Incidence of any OC in population	9	34	91	396	1, 2, 4, 6	8^a^
Friedman [26]		US; population-based	Cohort	4,413,100 patients	Incidence of any OC in population	68	762	361,802	4,050,468	NR	9^a^
Hippiseley-Cox [27]	Male	UK; population-based	Cohort	989,729, OC 1225	Incidence of any OC in population	216	120,866	1009	867,638	1, 4, 6	9^a^
	Female	UK; population-based	Cohort	1,013,565, OC 584	Incidence of any OC in population	78	104,670	506	908,311	1, 4, 6	9^a^
Vinogradava [28]		UK; population-based	Nested case control	16,200 patients OC 3159, control 13,041	Incidence of any OC in population	496	2106	2663	10,935	4, 6	9^a^
Bhutta [29] (abstract)		UK; population-based	Prospective cohort	18,484 patients OAC 581, OSC 322	Incidence of any OC in population	615	2539	3101	12,229	NR	7^a^
Lai [30]		Taiwan; population-based	Case control	2745 patients OC 549, control 2196	Incidence of any OC in population	49	238	500	1958	1, 2, 8, 9	6^a^
Chan [31]		Taiwan; population-based	Case control	985 patients OC 197, control 788	Incidence of any OC in population	29	131	168	657	8, 9	7^a^
Alexandre [32]	EAC	UK; population-based	Nested case control	OAC 581 Control 2167	Incidence of OAC in population	60	222	521	1945	4, 6, 9	8^a^
	EGJA			OAC 213 Control 783	Incidence of OAC in population	20	79	193	704	4, 6, 9	8^a^
Beales 2012b [33]		UK; hospital-based	Case control	560 patients OAC 112, control 448	Incidence of OAC in population	19	158	93	290	1, 2, 4, 5, 6, 9	8^a^

Variables adjusted for (1) age, (2) sex, (3) race, (4) smoking, (5) alcohol use, (6) obesity, (7) BO length, (8) oesophagitis or reflux symptoms, (9) medications (NSAIDs/aspirin)

*NR* not reported

^a^Statin use at baseline and not during course of study

### Outcomes Assessed

The primary analysis examined the association of statin and the incidence of oesophageal carcinoma as well as oesophageal adenocarcinoma specifically, through comparison of users and non-users. Subgroup analyses were used to investigate this further. Three categories were devised according to study cohort characteristics. These included OAC incidence in a population cohort, OAC incidence in Barrett’s oesophagus cohort, and incidence of all oesophageal cancers in a population cohort. Secondary analysis was focussed on examining these studies for any potential duration or dose relationship between statin and cancer incidence. In order to perform this, we limited analyses on duration or dose to studies that explicitly provided reliable statin use description data.

### Statistical Analysis

Review Manager (Revman) version 5.3 (Nordic Cochrane Center, Copenhagen, Denmark) was used to calculate the pooled risk ratio (compiling ORs or HR from individual studies) using the inverse variance method, random effects model as previously described [12]. Due to the relative rarity of outcomes, OR were considered as approximations of HR and RR. Analysis was carried out on unadjusted as well as adjusted risk ratios. Statistical heterogeneity was assessed using the Cochrane *I* ^2^ statistic, with *I* ^2^ > 25% indicating moderate statistical heterogeneity and *I* ^2^ > 50% indicating a substantial level of heterogeneity. A sensitivity analysis was performed by separately omitting one study at a time to assess if the pooled estimate had changed significantly compared to the results of all pooled studies.

## Results

**Fig. 1:**
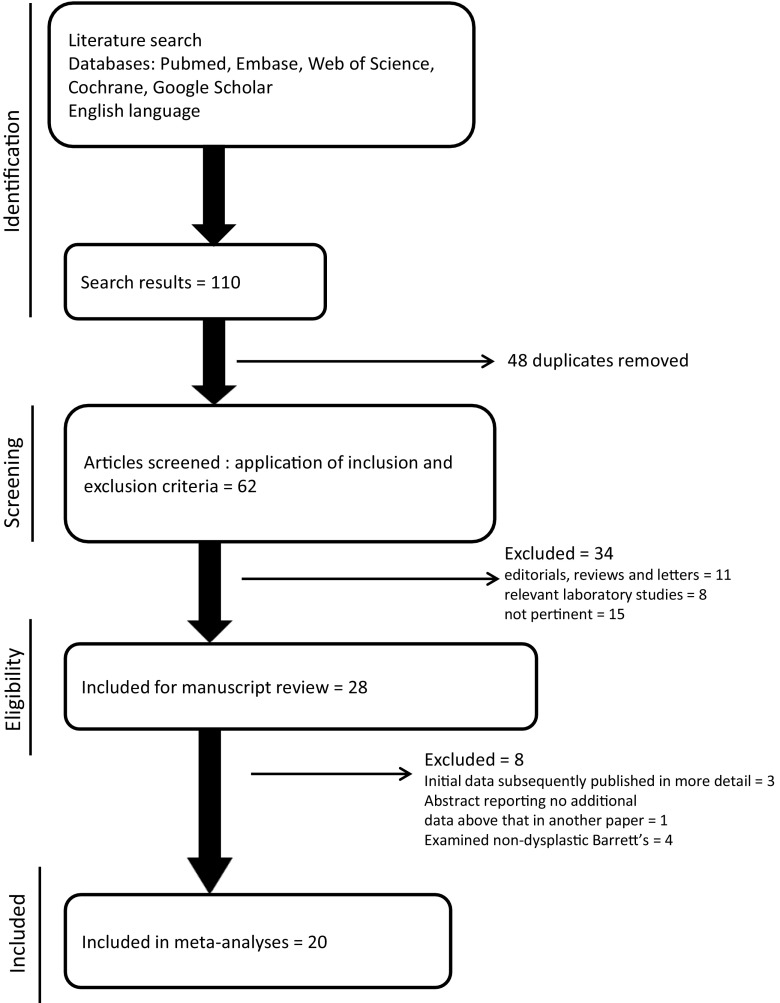
Flow chart showing process of study selection and data extraction

The search strategy identified 110 individual potentially suitable publications. Through the eligibility and screening processes, a total of 20 publications were included in the meta-analysis [14–33]. This totalled 22 different relevant studies as two publications reported separate data and adjusted odds ratios for different cohorts (either separate data for males and females or separate histological subtype of oesophageal cancer) [27, 32] (Fig. 1). All studies were published in English. The studies identified were clearly split into three categories based upon the specific population being examined: the association between statin use in the community and incidence of OAC, the relationship between statin use in a BO cohort on OAC rates, and the association with all oesophageal cancer on a population scale. Table 1 lists the studies included and their important characteristics. Two of the included studies were only published in abstract format; however, sufficient relevant information necessary for analysis was extracted from them [20, 29]. The other studies were in full peer-reviewed format.

For each planned subgroup analyses, separate meta-analyses were conducted on adjusted and unadjusted data. This was due to the heterogeneity in presentation of risk factor adjustment in the studies being analysed. As in the previous meta-analysis [12], heterogeneity in the reporting of statin dose and duration meant statistical analysis of this data would not be feasible.

### Characteristics of Included Studies

Twenty-two datasets reported on a total of 373,263 cases and 6,105,704 controls. As a proportion, 3.7% of the total population were statin users. Of 236,608 statin users, approximately 0.82% developed HGD/OC. Of 6,231,642 non-statin users, 5.90% developed HGD/OC. Details of the included studies are given in Table 1. The studies were from a variety of geographic locations with five from the USA and seven from the UK. No randomised control trials were identified during the search and in total, data were available from eight cohort, six nested case-control, and six case-control studies The majority of outcome assessments in nested case-control studies [23, 24, 32] and cohort study [19] were done through record linkage. Two case-control studies [18, 33] ascertained exposure through the use of structured interviewers where participants were not completely blind to case-control status. One case-control study analysed exposure through the use of secure records (i.e. medical notes). Eleven studies [14–24] examined statin use and the incidence of OAC in Barrett’s cohort. Eight datasets reported [25–31] the impact of statins on incidence of any OC in population and two studies (with a total of 3 datasets [32, 33]) looked at the relationship between statins and OAC in the general population.

Table 2 displays the baseline characteristics of the population from each study. Mean age at BO diagnosis or age of control in nested case-control studies ranged from 60.7 to 70.9 years. In case-control studies, the mean age of diagnosis of OC ranged from 60.9 to 70.7 years. With the exception of one study [32], the proportion of men as part of study population exceeded 60%. Length of BO was only reported in two studies [15, 16]. Three studies reported rates of reflux symptoms and endoscopic oesophagitis [16, 22, 31]. Statin use in the included study populations ranged from 13 to 54%. Proton pump inhibitors (PPI) used in the study populations ranged from 51 to 94%.

**Table 2:** Baseline characteristics of patients in studies included in the meta-analysis

a. EAC incidence in Barrett’s patients										
Study (reference) C: cohort C: case control nCC: nested case control	Cohort: age at BO diagnosis (years, mean (SD)) Case control: age of OAC/OC (corresponding control), years	Gender (% men)	Race (%Caucasian)	Obesity (% with BMI > 30)	Smoking (% ever)	Reflux symptoms or endoscopic oesophagitis	Length of BO (>3 cm)* (>2 cm)^	Other medication use		
								Aspirin NSAIDs (%)	Statins (%)	PPIs (%) current
Nguyen 2009 [16] C *N* = 344, 33 (HGD/EAC)	60.7 (11.8)	94.2	90.4	NR	NR	NR	29.1*	49.1	25.3	67.2
Nguyen 2010 [18] CC *N* = 812	65	97	74	NR	NR	NR	NR	57.6	46.4	94.8 93.8
Kastelein 2011 [20] C *N* = 570, 38 (HGD/EAC)	61 (53–68)	71	NR	19	19 (current)	29/9	100^	20.4	18.9	NR
Kantor 2012 [21] C	NDR	81.3	NR	NR	64	NR	NR	41.2 (current)	13.6	NR
Beales 2012a [22] CC	Case 68.9 (12.1) EAC Control 67.3 (12.0) BO	80	100	NR	67.1 59.4	N/A	N/A	21.1 33	20 35.3	N/A
Krishnamoorthi 2016 [26] C	63 (13.5)	62.6	NR	21.32	51.91	NR	NR	62.27	27.62	84.65
Iyer 2014 [25] abstrac C	63.04 (13.79)	63.89	NR	NR	NR	NR	NR	NR	NR	NR
Agrawal 2014 [27] CC	Case 65.9 (±9.5) EAC Control 61.4 (±11.4) BO	98	96	74	64.8	NR	NR	45	62.1	88.3
Masclee 2014 [28] nCC	UK 64.8 (SD 13.8) NL 61.2 (SD 13.4)	UK 63 NL 62	NR	NR	UK 51.7 NL 49.48	0.04	N/A	Aspirin: UK 48.93 NL 0.06 NSAIDs: UK 22.56 NL 13.5	UK 35.97 NL 16.33	UK 86.8 NL 51.66
Cooper 2014 [29] nCC	63 (52–72) BO	63	NR	NR	55	NR	NR	Aspirin 32 NSAIDs 68	1124 (30)	3569 (97)
Nguyen 2015 [32] nCC	Case 64.7 (9.1) Control 64.5 (9.1)	100 100	89.1 85.7	47 42.1	19 13.4	NR	NR	Aspirin 1, NSAID 23.8 Aspirin 1.3, NSAID 29.8	40.2 54	63 80.4
b. All oesophageal cancer incidence in population-based studies										
Study	Cohort: age at BO diagnosis, years Case control: age OC/corresponding control	Gender (% men)	Race (%Caucasian)	Obesity (% with BMI > 30)	Smoking (% ever)	Reflux symptoms or endoscopic oesophagitis	Length of BO	Other medication use		
								Aspirin/NSAIDs (%)	Statins (%)	PPIs (%) current
Kaye 2004 [31] CC	NR	NR	NR	NR	NR	NR	NR	NR	NR	NR
Friedman 2008 [15] C	NR								9.2 men 7.9 women	
Hippiseley-Cox 2010 (men) [17] C	NR	NR	NR	NR	NR	NR	NR	NR	NR	NR
Hippiseley-Cox 2010 (women) [17] C	NR	NR	NR	NR	NR	NR	NR	NR	NR	NR
Vinogradava 2011 [19] nCC	NR	NR	NR	NR	NR	NR	NR	NR	16	NR
Bhutta 2011 [33] C	NR	NR	NR	NR	NR	NR	NR	NR	16	NR
Lai 2012 CC [24]	Case 60.9 (12.9) Control 60.3 (13.3)	92.9 92.9	NR	0.55 0.18	1.64 1.68	NR	NR	34.1, 95.8 32.6, 92.0	8.93 10.8	47.7 12.9
Chan 2013 CC [34]	Case 63.96 (9.82) Control 63.94 (9.69)	88.14 88.32	NR	NR	NR	26.40/8.63 10.03/1.65	NR	NSAID 67.51 NSAID 77.79	14.7 16.6	NR
c. EAC development in population-based studies										
Study	Cohort: age at BO diagnosis, years Case control: age of OAC/corresponding control	Gender (% men)	Race (%Caucasian)	Obesity (% with BMI > 30)	Smoking (% ever)	Reflux symptoms or endoscopic oesophagitis	Length of BO	Other medication use		
								Aspirin/NSAIDs (%)	Statins (%)	PPIs (%) current
Beales 2012b [23] CC	Case 70.7 (10.3) Control 69.3 (12.6)	76 76	NR	NR	42.8 32.1	NR	NR	9.8/2.6 21.9/4.9	16.9 35.3	75 69
Alexandre 2014 [30] OAC	Case 70.7 (11.4) Control 70.6 (11.6)	77.8 77.6	NR	18.9 14.8	60.9 53.4	NR	NR	13.4/12.7 11.8/17.5	10.3 10.2	52.2 11.5
OGJA	Case 68.5 (11.9) Control 68.3 (12.1)	77 76.1		20.0 16.3	59.1 52.8			9.9/17.8 8.6/16	9.9 10.1	53.5 11.5
ESC nCC	Case 71.9 (12.4) Control 71.9 (12.3)	38.6 37.7		9.5 17.6	56.9 45			11.4/16.6 11.1/16	6.0 8.4	NR NR

* Barrett's segment > 3 cm, ^ Barrett's segment > 2 cm

*NR* not reported, *N/A* not available

### Quality Assessment

The studies included in the meta-analysis were ranked as being medium (11) to high quality (11) with the exception of two studies [20, 30]. A more detailed breakdown of study quality assessment can be found in Table 1. Baseline characteristics in patients are relatively consistent across studies producing an appropriate pooled population for oesophageal carcinoma. This was a population consisting of a majority Caucasian male population of age greater than 60 years. Overall 13 studies included adjusted for concomitant potentially chemopreventative medication in the form of aspirin/non-steroidal anti-inflammatory drugs (NSAIDs). The majority of studies also adjusted for two other main risk factors involved in the development of OC: smoking and obesity. However, adjustment for other risk factors was variable between studies. Due to the variability in correcting for and reporting potential confounders, the pooled data for both unadjusted and adjusted odds ratios were separately analysed.

### Statin Use in Barrett’s Cohorts Progressing to Adenocarcinoma

A total of 11 studies were included within this analysis. This included five cohort studies, two case-control studies, and four-nested case-control studies. The total sample included a minimum of 1057 cancer/HGD cases and 17,741 controls with non-cancerous with BO (the actual numbers included in one study are not available [20]). All studies adjusted for age and gender except for one study that did not adjust for gender [24]. Three studies adjusted for race [14, 16, 18]. Six studies adjusted for smoking [17–19, 23, 24, 33]. Only one study adjusted for alcohol use [18]. Four studies adjusted for obesity [17–19, 24]. Two studies adjusted for length of BO [15, 16] and reported reflux with/without endoscopic oesophagitis [16, 18]. Five studies adjusted for concomitant medication use (specifically NSAID/aspirin use) [14–16, 18, 22]. One study [15] did not report adjusted OR in the final publication and hence was not included in the relevant meta-analysis examining adjusted data. One study [19] used a time-varying model to assess HR. This was used to give a more reliable estimate, as it will take into account the time windows when the patients are taking or not taking statins. This study was incorporated into the adjusted OR model.

**Fig. 2:**
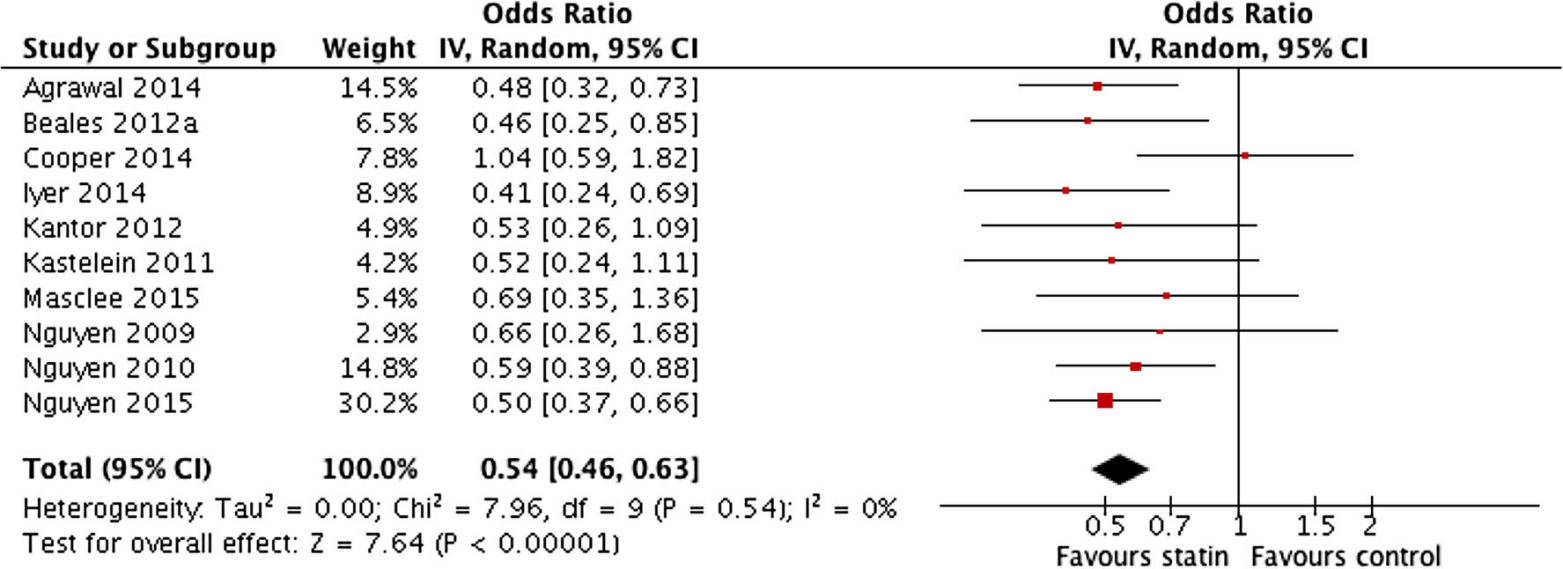
Meta-analysis of pooled unadjusted odds ratios for the effect of statin use on the development of oesophageal adenocarcinoma in patients with Barrett’s oesophagus

**Fig. 3:**
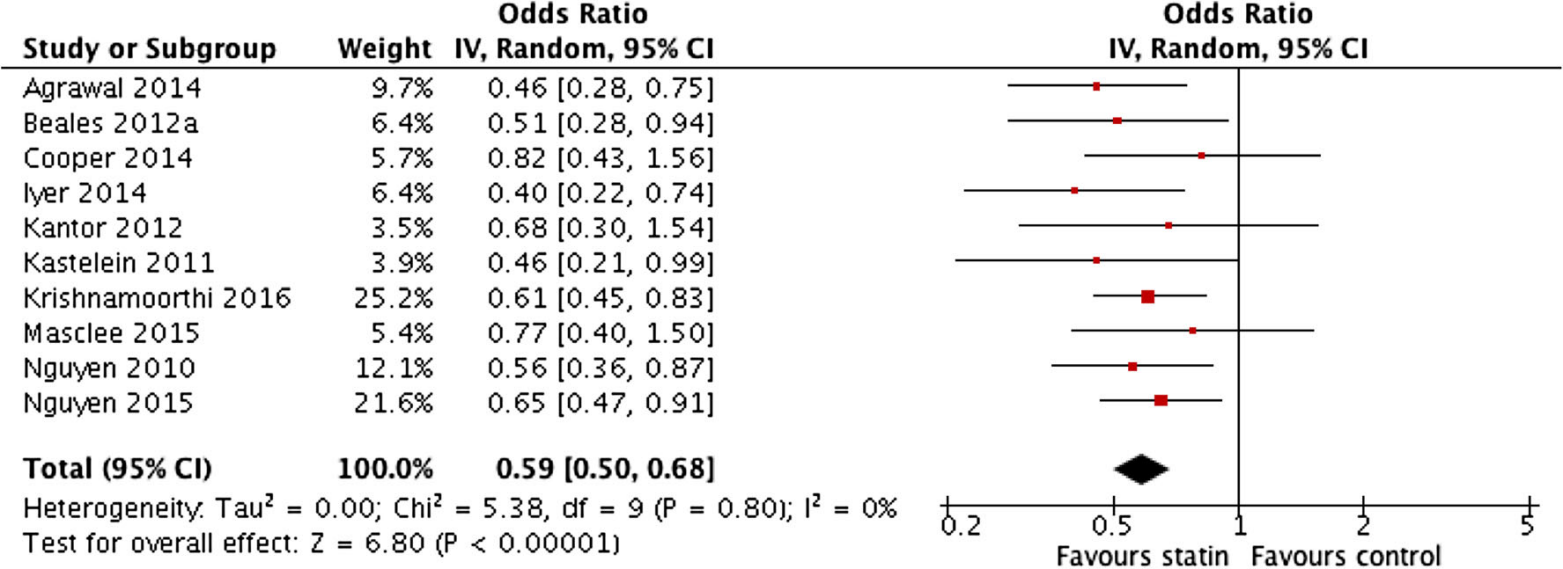
Meta-analysis of pooled adjusted odds ratios for the effect of statin use on the development of oesophageal adenocarcinoma in patients with Barrett’s oesophagus

The pooled unadjusted data showed a significant inverse association between statin use and the incidence of OAC in Barrett’s cohorts (pooled OR 0.54 (95% CI 0.46 to 0.63)) (*P* < 0.0001, *I* ^2^ = 0%) (Fig. 2). This confirmed the pooled adjusted ORs (OR 0.59 (95% CI 0.50 to 0.68)) (*P* < 0.00001, *I* ^2^ = 0%) (Fig. 3). There was no significant heterogeneity in the studies and sensitivity analyses showed that the omission of any single study had no effect on the overall results. Both case-control and cohort studies produced very similar results and the results of the study only presented in abstract form for which less detail is available were consistent with all the other studies [20].

There was major variation in the reporting of duration and dose of statin use within the included studies. This outcome was therefore unsuitable for statistical analysis. Nguyen et al. [14] suggested that duration of statin use greater than 1 year was associated with a reduced incidence of OAC (aOR 0.52; 95% CI 0.3 to 0.91). Beales et al. [22] reported that statin use of greater than 5 years was associated with a reduced incidence of OAC (aOR 0.41; 95% CI 0.15 to 0.85). Nguyen et al. [24] also reinforced this general finding with both unadjusted and adjusted OR for the period of 6 to 18 months showing a reduced incidence of OAC (OR 0.41; 95% CI 0.26 to 0.63, and aOR 0.52; 95% CI 0.32 to 0.85). Although other ORs within this study [24] showed efficacy at less than 6 months and greater than 18 months, these ORs were not statistically significant after adjusting for confounders and potential risk factors. In terms of dosage, Beales et al. [18] reported that statin dose above 40 mg equivalent of simvastatin was associated with a more pronounced inverse association than doses below 40 mg simvastatin dose equivalents (adjusted OR 0.31; 95% CI 0.05 to 0.97 compared to 0.59; 95% CI 0.27 to 0.98, respectively). The other studies did not provide data on statin dose, type, or duration.

### Statin Use in Population Cohort Progressing to Adenocarcinoma

This subgroup analysis consisted of data extracted from two publications. One paper reported separate data from OAC and oesophagogastric junction adenocarcinoma (OGJA); due to the potential difference in biology, these were analysed and reported separately [32]. All studies were case-control studies. The total sample size was 4305 patients. This consisted of 907 cases of OAC with 3398 controls. All studies included adjusted and unadjusted data, and hence, both sets of data were compiled into the meta-analysis. Alexandre et al. [32] adjusted for risk factors such as smoking, obesity, and concomitant medication use in the form of NSAIDs, aspirin, and PPIs. Beales et al. [33] adjusted for age, gender, smoking, alcohol, obesity, and medications.

**Fig. 4:**
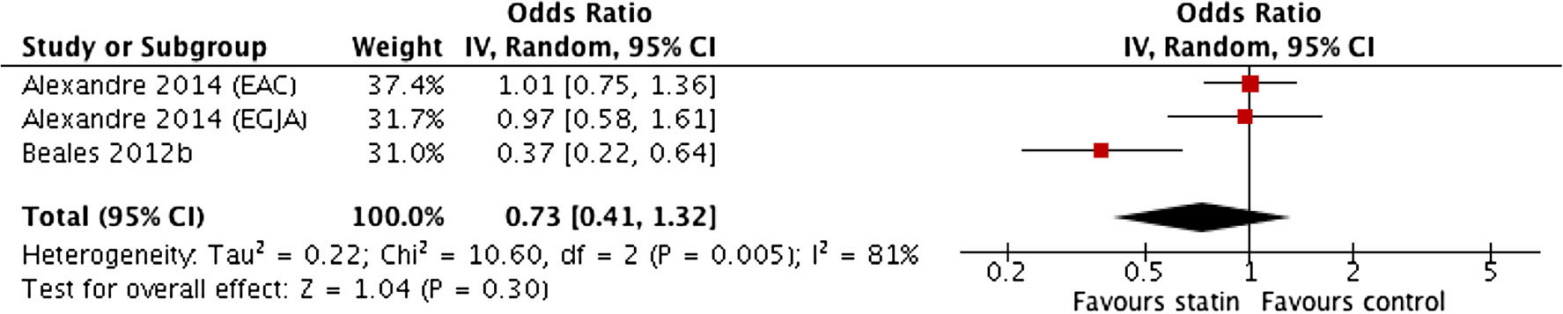
Meta-analysis of pooled unadjusted odds ratios for the effect of statin use on the development of oesophageal adenocarcinoma in population-based studies

**Fig. 5:**
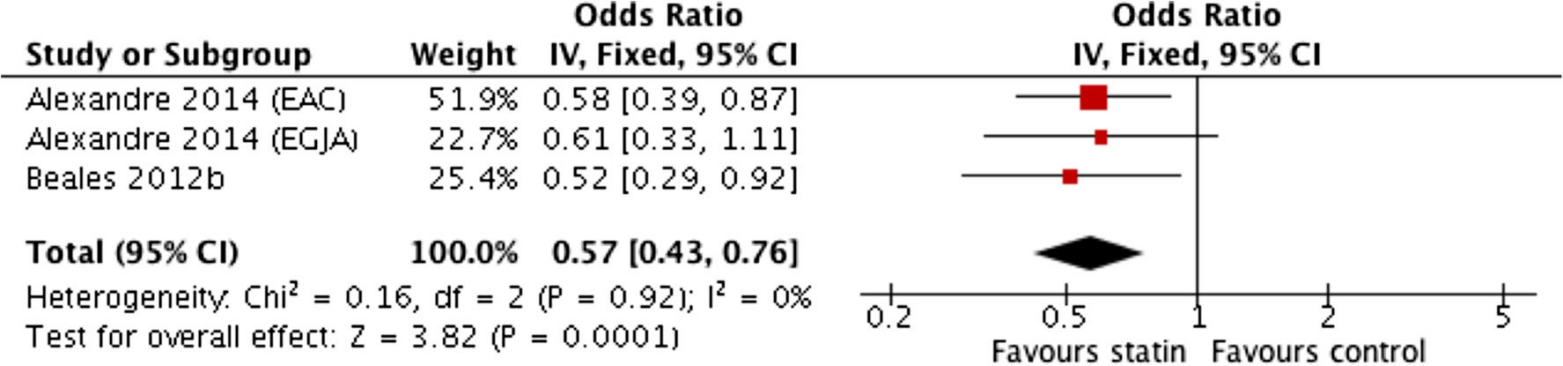
Meta-analysis of pooled adjusted odds ratios for the effect of statin use on the development of oesophageal adenocarcinoma in population-based studies

Pooled unadjusted ORs showed a non-significant association with wide confidence intervals with significant heterogeneity (0.73 (0.41–1.32)) (*I* ^2^ = 81%) (Fig. 4). However, the meta-analysis of pooled adjusted data (Fig. 5) showed a significant negative association between statin use and OAC incidence (OR 0.57; 95% CI 0.43 to 0.76) (*P* < 0.001), with no significant heterogeneity between studies (*I* ^2^ = 0%).

When examining duration of statin use, Beales et al. [33] showed duration of use greater than 5 years had greater inverse association (adjusted OR 0.29; 95% CI 0.1 to 0.67) compared to other time periods such as 2 to 5 years (adjusted OR 0.51; 95% CI 0.19 to 0.97). Both studies [32, 33] examined dose with a common dose margin of 40 mg simvastatin. Beales et al. [33] reported that doses greater than 40 mg simvastatin were associated with a greater reduction in risk (adjusted OR 0.16; 95% CI 0.05 to 0.6) than doses less than 40 mg (adjusted OR 0.53; 95% CI 0.25 to 0.86). Although the data provided for statin dose less than 40 mg in Alexandre et al. [32] are not statistically significant, the statin dose for greater than 40 mg shows a substantial reduction in risk of OAC in a population cohort (adjusted OR 0.51; 95% CI 0.27 to 0.96).

### Statin use in Population Cohort Progressing to Oesophageal Cancer (All Types)

This subgroup analysis examined the potential effect of statins on the incidence of all types of oesophageal carcinomas in population cohorts. A total of eight cohorts were used in this analysis. This consisted of seven studies with one study [27] providing two populations consisting of the male and female population analysed separately. The sample size totalled 6,455,338 patients. There were 371,400 cases of oesophageal carcinomas in a background of 6,083,938 patients from population cohorts. Three studies adjusted for age [25, 27, 30], smoking [25, 27, 28], and obesity [25, 27, 28]. Two studies adjusted for gender [25, 30]. Lai et al. [30] and Chan et al. [31] both adjusted for symptomatic reflux and endoscopic oesophagitis along with concomitant use of medications (aspirin/NSAIDs). One study [26] did not provide details of any adjustment in both study design and, statistically, hence was unable to be included in the adjusted OR meta-analysis.

**Fig. 6:**
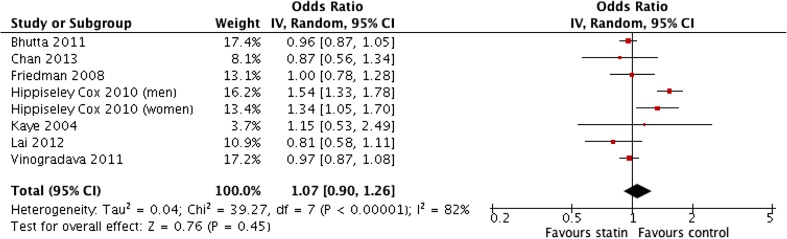
Meta-analysis of pooled unadjusted odds ratios for the effect of statin use on the development of all types of oesophageal carcinoma in population-based studies

**Fig. 7:**
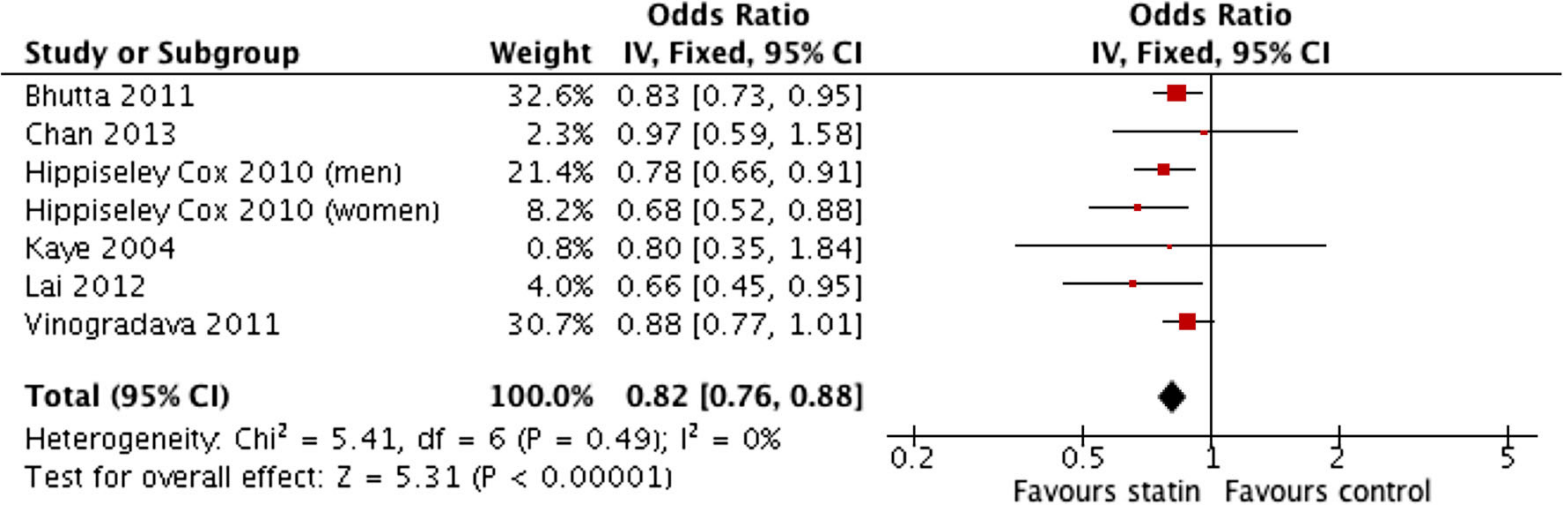
Meta-analysis of pooled adjusted odds ratios for the effect of statin use on the development of all types of oesophageal carcinoma in population-based studies

The result of the meta-analysis of unadjusted odds ratios (1.07 (0.90–1.26)) (Fig. 6) was statistically insignificant. This was largely due to the influence of two studies [27, 25]. The unadjusted and adjusted ORs within the study by Hippiseley Cox et al. [27] were considerably different. There was substantial heterogeneity in the pooled unadjusted data (*I* ^2^ levels 82%). The pooled analysis of adjusted ORs demonstrated that the use of statins in a population cohort was associated with a significantly lower incidence of all oesophagus (OR 0.82; 95% CI 0.76 to 0.88) (*P* < 0.00001) with no heterogeneity in the data (*I* ^2^ = 0%) (Fig. 7)*.* Sensitivity testing showed that the significance of the result was not influence by the exclusion of any single study.

Similar to the previous analysis, there was significant heterogeneity in the methods of data collection and reporting regarding duration and dosage of statins. This rendered statistical analysis inappropriate. Friedman et al. [26] actually reported statins were associated with an increased risk of OC in the male population (adjusted OR 1.7; 95% CI 1.05 to 2.75). However, with such large confidence intervals in place, this data are potentially unreliable. Data from Lai et al. [30] showed that statins were associated with a reduced incidence of OAC in a population cohort particularly when used for greater than 12 months (adjusted OR 0.14; 95% CI 0.04 to 0.56). In terms of statin doses, only Hippiseley-Cox et al. [27] provided detailed information. In males, doses of simvastatin equivalent to 40–80 mg showed an aOR 0.66; 95% CI 0.48 to 0.91 with the incidence of all oesophageal cancers. Doses of 10 mg atorvastatin were associated with reduced incidence of OC (aOR 0.68; 95% CI 0.49 to 0.95). In females, this trend was reversed with data suggesting dosages of 10 mg atorvastatin (aOR 0.67; 95% CI 0.49 to 0.95) were more effective in reducing OC rates in a population cohort than 40–80 g simvastatin (aOR 0.82; 95% CI 0.66 to 0.91).

## Discussion

The primary objective of this meta-analysis was to examine association of statins with the development of oesophageal cancers, particularly oesophageal adenocarcinoma. Our pooled estimates show that statins have a strong association with a reduction in the risk of OAC (approximately 41% reduction of risk) particularly when used in patients with pre-existing Barrett’s oesophagus. The adjusted data from 11 studies provides a reasonably narrow confidence interval for this figure. These data are similar to those reported in the previous meta-analyses: Singh et al. [11] and Beales et al. [12] adjusted OR = 0.59; 95% CI, 0.45–0.78 and adjusted OR = 0.57; 95% CI, 0.43–0.75, respectively. The current updated meta-analysis has more than doubled the number of studies included (from 5 to 11) and increased the number of OAC cases 3-fold (317 to 1057), and this inclusion of additional studies has allowed a much tighter definition of the likely margin of association with much smaller confidence intervals.

A similar degree of reduction in OAC was associated with statin use in population cohorts. The pooled adjusted odds ratios showed a reduction of 43% in OAC incidence. However, with only three datasets included, it is relatively the most underpowered of all analyses included in this study. However, this is a new finding; previous systematic reviews did not allow statistical analysis of this particular subgroup. Further studies are warranted to investigate this effect more thoroughly especially in light of the rising incidence rates of OAC. It is notable that this subgroup analysis included a specific oesophagogastric junction adenocarcinoma group, whilst there may be some important biological differences between this and more classical OAC [8], it was felt appropriate to include this cohort as the majority of the other studies included in this systematic review included junctional cancers within the cancer cases, making no distinction between the OAC and OGJA for statistical analysis. Two studies [18, 33] specifically excluded Siewert III OGJA cancer.

It remains an area of active research whether statins have beneficial anti-cancer effects merely on progression of non-dysplastic BO or whether they are actually associated with a reduction in the development of BO itself. Whilst most of the in vitro studies have examined the effects of statins on various models of Barrett’s oesophagus, recent data including a meta-analysis have reported that statin use is associated with a reduced incidence Barrett’s oesophagus [35].

Statin use in the population is associated with a reduced incidence (by 18%) of all types of oesophageal cancers. Again, three of the major studies [27–29] included in the analysis provided very consistent data in describing this effect. The previous meta-analysis [12] suggested very similar reduction of risk at OR = 0.81; 95% CI 0.75–0.88. There was much more heterogeneity in the unadjusted results examining statin use at the population level and all oesophageal cancers than in the other analyses. The heterogeneity was significantly reduced when comparing the odds ratios adjusted for other variables. This probably reflects the wider variety of geographical and health care locations included in this group (certainly compared to the more homogeneous secondary care Barrett’s cohorts) and reinforces the problems with potential confounding by indications and risk factors in studies at that population level. Despite this, there were no obvious outlying studies and the pooled analysis of the adjusted odds ratios showed no significant heterogeneity.

Most of the published data have examined the relationship between statins and OAC; there are much less data concerning squamous cancer. Although there are well-performed laboratory studies providing biological plausibility and exploring the effects of statins on oesophageal adenocarcinoma and non-malignant Barrett’s cells [6, 7, 10], these corroborating studies are lacking as regards oesophageal squamous cancer. Given the contrasting biology and epidemiology between oesophageal adenocarcinoma and squamous cancers, it would not be surprising if the effect of statins differed between the two. The relatively smaller apparent protective effect against all oesophageal cancers combined compared to specifically against OAC may imply that statins have less effect on the development of squamous cancer but further studies are indicated. The relative and absolute numbers of adenocarcinomas and squamous cancers within the studies examined in this population-level data meta-analysis are unknown and, given the disparity of geographical locations studies, are likely to vary between studies, further contributing to the heterogeneity of results in this subgroup analysis. We acknowledge that these population-level, all-oesophagal cancer data are the weakest and least informative of those specifically included in this systematic review, but feel these data should be included for completeness and to stimulate further research into any associations between statin use and squamous cancer. However, the pooled data on all oesophageal cancers are still consistent with statin use being associated with a lower risk of oesophageal adenocarcinoma.

The main limitation to this meta-analysis is that all included studies are observational (i.e. cohort or case-control). As such, regardless of the sample size involved in this study, it is not completely free of bias and despite the generally high quality of the observational studies included in this systematic review, uncorrected bias may still influence the results. However, the consistency of the results, including lack of heterogeneity, from different geographical locations, health care systems, and research methodologies suggests that unadjusted bias is unlikely to have a major effect on our findings, although it would be important to establish what the association is between statin use and cancer risk in relation to specific other risk factors for OAC such as smoking, obesity, and visceral obesity [8]. In the adenocarcinoma-specific analysis, particularly in the Barret’s cohorts, the minimal difference between the pooled adjusted and unadjusted ORs, suggests that confounding by indication and residual bias, are unlikely to be contributing to the results.

It was notable that the number and variety of potential cofounding variables adjusted varied considerably between the studies and this would seem to be a major focus for future work. Despite the evidence that proton pump inhibitors and NSAIDs [33, 36] are also associated with a reduced incidence of OAC, not all studies provided data to enable accurate adjustment. Laboratory studies and our previous meta-analysis showed that the combination of statins and cyclo-oxygenase inhibitors appeared to be additive in both effects on cell lines [6, 7] and in reducing the incidence of OAC (pooled aOR 0.26 (0.1–0.68)) [12]. However, the more recent studies have not examined or reported the data to enable further verification of this association and it is recommended that any further studies specifically examine this.

Probably the weakest aspect of the current study is the lack of data on statin type (lipophilic or hydrophilic), dose, drug, or duration. Previous meta-analyses [11, 12] were similarly limited in this regard but unfortunately despite the increase in the number of studies and subjects included in the systematic review, the data are still inadequate to draw any firm conclusions. Most studies did not specifically examine this aspect of statin use and when it was reported, the categories used were too heterogeneous for statistical analysis. Where reported there seemed to be a tendency for higher doses and longer durations to be associated with a lower incidence of OAC but any future studies should specifically address these issues.

In conclusion, our updated systematic review and meta-analysis show that statin use is associated with a significantly reduced incidence of oesophageal adenocarcinoma in both Barrett’s cohorts (by 41%) and population-based studies (by 43%). There is also a reduced incidence of all oesophageal cancers in population-based studies (by 18%), although it is possible that this is driven mainly through a reduction in the incidence of OAC rather than OSC; that hypothesis requires further investigation. The inclusion of additional studies has enabled much tighter precision of the estimate of the reduction of OAC associated with statin use in Barrett’s cohorts.

The consistency of the results across multiple studies combined with supportive experimental evidence does suggest that statins may have useful chemopreventative effects in Barrett’s oesophagus. Given the relatively low rate of neoplastic progression in BO (approximately 1 in 300 per year [37, 38]), it may not be cost-effective to merely use statins as chemopreventative agents, but given that the main cause of death in patients with BO is not oesophageal cancer but vascular diseases, it may be prudent to ensure that statins that are definitely prescribed to patients with Barrett’s oesophagus were indicated by vascular risk [39].

## Electronic Supplementary Material

ESM 1 (DOCX 12 kb)

## Abbreviations

aOR: Adjusted odds ratio
BO: Barrett’s oesophagus
CI: Confidence interval
OAC: Oesophageal adenocarcinoma
OGJA: Oesophagogastric junction adenocarcinoma
OSC: Oesophageal squamous cancer
NSAID: Non-steroidal anti-inflammatory drug
OR: Odds ratio

## References

[1] Pennathur A, Gibson MK, Jobe BA, Luketich JD (2013). Oesophageal carcinoma. Lancet.

[2] Arnold M, Soerjomataram I, Ferlay J, Forman D (2015). Global incidence of oesophageal cancer by histological subtype in 2012. Gut.

[3] Wang Z, Goodman M, Saba N, El-Rayes BF (2013). Incidence and prognosis of gastroesophageal cancer in rural, urban, and metropolitan areas of the United States. Cancer.

[4] Zhang Y (2013). Epidemiology of esophageal cancer. World J Gastroenterol.

[5] Shaheen NJ, Overholt BF, Sampliner RE, Wolfsen HC, Wang KK, Fleischer DE, Sharma VK, Eisen GM, Fennerty MB, Hunter JG (2011). Durability of radiofrequency ablation in Barrett’s esophagus with dysplasia. Gastroenterology.

[6] Ogunwobi OO, Beales IL (2008). Statins inhibit proliferation and induce apoptosis in Barrett’s esophageal adenocarcinoma cells. Am J Gastroenterol.

[7] Fang D, Das KM, Cao W, Malhotra U, Triadafilopoulos G, Najarian RM, Hardie LJ, Lightdale CJ, Beales IL, Felix VN (2011). Barrett’s esophagus: progression to adenocarcinoma and markers. Ann N Y Acad Sci.

[8] Long E, Beales IL (2014). The role of obesity in oesophageal cancer development. Ther Adv Gastroenterol.

[9] Alexandre L, Long E, Beales IL (2014). Pathophysiological mechanisms linking obesity and esophageal adenocarcinoma. World J Gastrointest Pathophysiol.

[10] Konturek PC, Burnat G, Hahn EG (2007). Inhibition of Barrett’s adenocarcinoma cell growth by simvastatin: invollvment of COX-2 and apoptosis-related proteins. J Physiol Pharmacol.

[11] Singh S, Singh AG, Singh PP, Murad MH, Iyer PG (2013). Statins are associated with reduced risk of esophageal cancer, particularly in patients with Barrett’s esophagus: a systematic review and meta-analysis. Clin Gastroenterol Hepatol.

[12] Beales IL, Hensley A, Loke Y (2013). Reduced esophageal cancer incidence in statin users, particularly with cyclo-oxygenase inhibition. World J Gastrointest Pharmacol Ther.

[13] Shamseer L, Moher D, Clarke M, Ghersi D, Liberati A, Petticrew M, Shekelle P, Stewart LA (2015). Preferred reporting items for systematic review and meta-analysis protocols (PRISMA-P) 2015: elaboration and explanation. BMJ.

[14] Nguyen DM, Richardson P, El-Serag HB (2010). Medications (NSAIDs, statins, proton pump inhibitors) and the risk of esophageal adenocarcinoma in patients with Barrett’s esophagus. Gastroenterology.

[15] Nguyen DM, El-Serag HB, Henderson L, Stein D, Bhattacharyya A, Sampliner RE (2009). Medication usage and the risk of neoplasia in patients with Barrett’s esophagus. Clin Gastroenterol Hepatol.

[16] Kastelein F, Spaander MC, Biermann K, Steyerberg EW, Kuipers EJ, Bruno MJ (2011). Nonsteroidal anti-inflammatory drugs and statins have chemopreventative effects in patients with Barrett’s esophagus. Gastroenterology.

[17] Kantor ED, Onstad L, Blount PL, Reid BJ, Vaughan TL (2012). Use of statin medications and risk of esophageal adenocarcinoma in persons with Barrett’s esophagus. Cancer Epidemiol Biomark Prev.

[18] Beales IL, Vardi I, Dearman L (2012). Regular statin and aspirin use in patients with Barrett’s oesophagus is associated with a reduced incidence of oesophageal adenocarcinoma. Eur J Gastroenterol Hepatol.

[19] Krishnamoorthi R, Borah B, Heien H, Das A, Chak A, Iyer PG (2016). Rates and predictors of progression to esophageal carcinoma in a large population-based Barrett’s esophagus cohort. Gastrointest Endosc.

[20] Iyer Prasad G., Borah Bijan J., Heien Herbert, Chak Amitabh (2014). 705 Rates and Predictors of Progression to Adenocarcinoma in a Large Population Based Barrett's Esophagus Cohort. Gastroenterology.

[21] Agrawal S, Patel P, Agrawal A, Makhijani N, Markert R, Deidrich W (2014). Metformin use and the risk of esophageal cancer in Barrett esophagus. South Med J.

[22] Masclee GM, Coloma PM, Spaander MC, Kuipers EJ, Sturkenboom MC (2015). NSAIDs, statins, low-dose aspirin and PPIs, and the risk of oesophageal adenocarcinoma among patients with Barrett’s oesophagus: a population-based case-control study. BMJ Open.

[23] Cooper S, Menon S, Nightingale P, Trudgill NJ (2014). Risk factors for the development of oesophageal adenocarcinoma in Barrett’s oesophagus: a UK primary care retrospective nested case-control study. United European Gastroenterol J.

[24] Nguyen T, Duan Z, Naik AD, Kramer JR, El-Serag HB (2015). Statin use reduces risk of esophageal adenocarcinoma in US veterans with Barrett’s esophagus: a nested case-control study. Gastroenterology.

[25] Kaye JA, Jick H (2004). Statin use and cancer risk in the General Practice Research Database. Br J Cancer.

[26] Friedman GD, Flick ED, Udaltsova N, Chan J, Quesenberry CP, Habel LA (2008). Screening statins for possible carcinogenic risk: up to 9 years of follow-up of 361,859 recipients. Pharmacoepidemiol Drug Saf.

[27] Hippisley-Cox J, Coupland C (2010). Unintended effects of statins in men and women in England and Wales: population based cohort study using the QResearch database. BMJ.

[28] Vinogradova Y, Coupland C, Hippisley-Cox J (2011). Exposure to statins and risk of common cancers: a series of nested case-control studies. BMC Cancer.

[29] Bhutta H Y, Alexandre L, Clark A, Holt S, Lewis M, Hart A (2012). PWE-008 Do statins prevent the histological subtypes of oesophageal cancer? Prospective data from the UK general practice research database (GPRD). Gut.

[30] Lai S, Liao K, Lai H, Muo C, Sung F (2012). Atorvastatin correlates with decreased risk of esophageal cancer: a population-based case-control study from Taiwan. Libyan J Med.

[31] Chan TF, Chiu HF, Wu CH, Lin CL, Yang CY (2013). Statin use and the risk of esophageal cancer: a population-based case-control study. Expert Opin Drug Saf.

[32] Alexandre L, Clark AB, Bhutta HY, Holt S, Lewis MP, Hart AR (2014). Statin use is associated with reduced risk of histologic subtypes of esophageal cancer: a nested case-control analysis. Gastroenterology.

[33] Beales I, Vardi I, Dearman L, Broughton T (2013). Statin use is associated with a reduction in the incidence of esophageal adenoncarcinoma: a case-control study. Dis Esophagus.

[34] Stroup DF, Berlin JA, Morton SC, Olkin I, Williamson GD, Rennie D, Moher D, Becker BJ, Sipe TA, Thacker SB (2000). Meta-analysis of observational studies in epidemiology: a proposal for reporting. Meta-analysis Of Observational Studies in Epidemiology (MOOSE) group. JAMA.

[35] Beales IL, Dearman L, Vardi I, Loke Y (2016). Reduced risk of Barrett’s esophagus in statin users: case-control study and meta-analysis. Dig Dis Sci.

[36] Singh S, Garg SK, Singh PP, Iyer PG, El-Serag HB (2014). Acid-suppressive medications and risk of oesophageal adenocarcinoma in patients with Barrett’s oesophagus: a systematic review and meta-analysis. Gut.

[37] Hvid-Jensen F, Pedersen L, Drewes AM, Sorensen HT, Funch-Jensen P (2011). Incidence of adenocarcinoma among patients with Barrett’s esophagus. N Engl J Med.

[38] Desai TK, Krishnan K, Samala N, Singh J, Cluley J, Perla S, Howden CW (2012). The incidence of oesophageal adenocarcinoma in non-dysplastic Barrett’s oesophagus: a meta-analysis. Gut.

[39] Moayyedi P, Burch N, Akhtar-Danesh N, Enaganti SK, Harrison R, Talley NJ, Jankowski J (2008). Mortality rates in patients with Barrett’s oesophagus. Aliment Pharmacol Ther.

