# Supplementary material for: Systematic Review and Meta-analysis: Use of Statins Is Associated with a Reduced Incidence of Oesophageal Adenocarcinoma
Source: J Gastrointest Cancer. 2017 Jul 10;49(4):442–54. doi: 10.1007/s12029-017-9983-0 (PMC6208835; doi:10.1007/s12029-017-9983-0)
Supplement: Supplementary file 1 — (DOCX 12 kb) [file 12029_2017_9983_MOESM1_ESM.docx]

Supplementary Table 1

Reporting of data In the meta-analysis as per JAMA 2008, 283: 2008-2012

| **Reporting of background** |  |
| --- | --- |
| Problem definition | Pages 1-2 |
| Hypothesis statement | Page 2 |
| Description of study outcomes | Page 4 |
| Type of exposure of intervention | Page 4 |
| Type of study designs used | Page 4 |
| Study population | Page 4 |
| **Reporting of search strategy** |  |
| Qualifications of searchers | Page 5 |
| Effort to include all studies and contact with authors | Page 5 |
| Databases and registries searched | Pages 4-5 |
| Search software | Pages 4-5 |
| Use of hand searching | Pages 4-5 |
| List of citations and exclusions | Tables 1 & 2 |
| Methods of dealing with non-English articles | N/A |
| Handling of abstracts | Page 7 |
| Description of contact with authors | N/A |
| **Reporting of Methods** |  |
| Description of relevance of studies | Tables 1 and 2 |
| Rationale for selection of data | Page 4-5 |
| Documentation of data classification and coding | Pages 4-6 |
| Assessment of confounding | Table1, pages 7-12 |
| Assessment of study quality | Table 1, pages 8-9 |
| Assessment of heterogeneity | Figures 2-7 |
| Description of statistical models | Pages 4 and 6 |
| Provision of tables and graphics | Tables 1 and 2, Figures 2-7 |
| **Reporting of results** |  |
| Graphic summarizing individual study estimates | Figures 2-7 |
| Table giving descriptive information about each study | Tables 1 and 2 |
| Results of sensitivity testing | Pages 9-12 |
| Indication of statistical uncertainty | Figures 2-7, pages 9-12 |
| **Reporting of discussion** |  |
| Quantitive assessment of bias | Pages 13-14 |
| Justification for exclusion | N/A |
| Assessment of quality of included studies | Table 1 and page 7 |
| **Reporting of conclusions** |  |
| Consideration of alternative explanation of results | Pages 13-15 |
| Generalization of results | Pages 13-15 |
| Guidelines for future research | Pages 14-15 |
| Disclosure of funding source | Page 17 |
